# Hedgehog Signaling Pathway in Fibrosis and Targeted Therapies

**DOI:** 10.3390/biom14121485

**Published:** 2024-11-22

**Authors:** Yuchen Hu, Linrui Peng, Xinyu Zhuo, Chan Yang, Yuwei Zhang

**Affiliations:** 1 Department of Endocrinology and Metabolism, West China Hospital, Sichuan University, Chengdu 610041, China; huyuchen9@stu.scu.edu.cn (Y.H.); linruipeng@stu.scu.edu.cn (L.P.); zhuoxy@stu.scu.edu.cn (X.Z.); 2 Center for Diabetes and Metabolism Research, West China Hospital, Sichuan University, Chengdu 610041, China; 3 Division of Endocrinology and Metabolism, State Key Laboratory of Biotherapy, West China Hospital, Sichuan University and Collaborative Innovation Center of Biotherapy, Chengdu 610041, China; yc199207@126.com

**Keywords:** hedgehog signaling pathway, fibrosis, targeted therapies

## Abstract

Hedgehog (Hh) signaling is a well-established developmental pathway; it is crucial for early embryogenesis, cell differentiation, and damage-driven regeneration. It is being increasingly recognized that dysregulated Hh signaling is also involved in fibrotic diseases, which are characterized by excessive extracellular matrix deposition that compromises tissue architecture and function. As in-depth insights into the mechanisms of Hh signaling are obtained, its complex involvement in fibrosis is gradually being illuminated. Notably, some Hh-targeted inhibitors are currently under exploration in preclinical and clinical trials as a means to prevent fibrosis progression. In this review, we provide a concise overview of the biological mechanisms involved in Hh signaling. We summarize the latest advances in our understanding of the roles of Hh signaling in fibrogenesis across the liver, kidneys, airways, and lungs, as well as other tissues and organs, with an emphasis on both the shared features and, more critically, the distinct functional variations observed across these tissues and organs. We thus highlight the context dependence of Hh signaling, as well as discuss the current status and the challenges of Hh-targeted therapies for fibrosis.

## 1. Introduction

The Hedgehog (Hh) signaling pathway was initially described regarding the developmental patterning of *Drosophila* in 1980 [1]. Published work demonstrates that Hh signaling, as a regulator of cell proliferation, differentiation, and tissue polarity, plays a crucial role in early embryonic development and tissue homeostasis and repair [2]. Although the transient activation of the Hh pathway is required for a wound-healing response, its prolonged activation is believed to promote excessive extracellular matrix (ECM) deposition, which drives progressive fibrosis in various tissues and organs [3,4]. Given its vital role in human fibrotic diseases, the targeting of Hh signaling to halt fibrosis progression is imperative and represents a promising therapeutic strategy. Notably, several Hh-targeted therapies have been advanced into clinical trials. This review provides a brief overview of the biological processes involved in Hh signaling. We consider up-to-date studies and summarize the roles of Hh signaling in fibrogenesis across the liver, kidneys, airways, and lungs, as well as other tissue types, focusing on both the commonalities and, more importantly, the differences in its function across these. We emphasize the context-dependent and tissue-specific characteristics of Hh signaling and discuss the current and potential antifibrotic therapies targeting Hh signaling, along with the challenges that remain.

## 2. Hh Signaling Pathway

**Figure 1:**
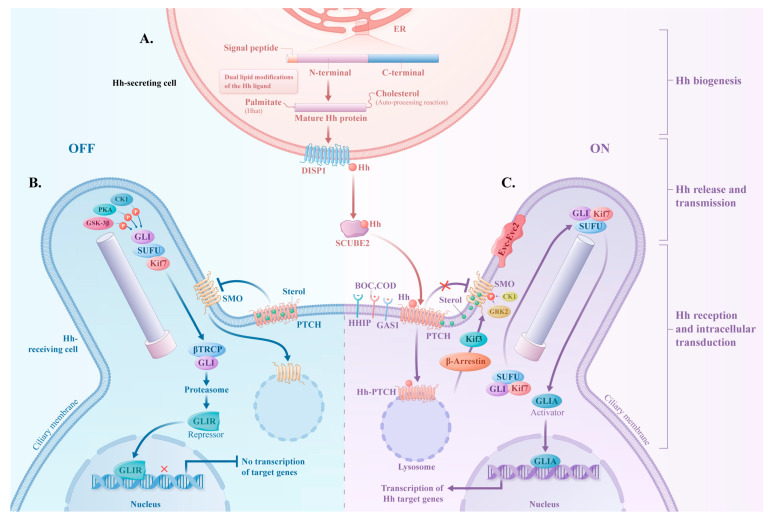
Biological processes of the canonical Hedgehog (Hh) signaling pathway. The major steps in the canonical Hh signaling pathway in mammals have been elucidated: Hh biogenesis, release, transmission, reception, and intracellular transduction. (**A**). In the endoplasmic reticulum (ER), the Hh ligand undergoes two sequential lipid modifications, including cholesterol added by an autoprocessing reaction and palmitate attached by Hh acetyltransferase (Hhat). Dispatched homologue 1 (DISP1) then transfers the Hh ligand to its carrier, the cubulin (CUB) domain of epidermal growth factor (EGF)-like protein 2 (SCUBE2). This SCUBE2-mediated soluble complex enables the long-range transport of the hydrophobic Hh ligand in the intercellular matrix to the Hh-receiving cell. (**B**). In the absence of the Hh ligand (pathway-off), patched (PTCH), located on the primary cilium (PC), inhibits Smoothened (SMO) as a sterol transporter, which means sterols are barely accessible to SMO. As a result, SMO remains inactive in the cytoplasm. Simultaneously, glioma-associated oncogene (GLI) interacts with the suppressor of fused (SUFU) to form a complex, which undergoes phosphorylation by protein kinase A (PKA), glycogen synthase kinase-3 (GSK3β), and casein kinase 1 (CK1). This is followed by ubiquitin-mediated proteolysis via β-transducin repeat-containing protein (βTRCP), producing a truncated repressor form of GLI (GLIR), which inhibits the transcription of target genes. (**C**). In the presence of the Hh ligand (pathway-on), the binding of the Hh ligand to PTCH is facilitated via co-receptors such as those cell adhesion molecule-related/downregulated by oncogenes (CDO), brother of CDO (BOC), and growth arrest-specific protein 1 (GAS1). The Hh-PTCH compound then leaves the ciliary membrane and is degraded, which can generate the environment where SMO can be accessible to sterols and be activated. Kinesin protein 3 (Kif3) and β-arrestin mediate the movement of SMO from the cytoplasm into the PC. CK1 and G protein-coupled receptor kinase 2 (GRK2) phosphorylate SMO to be fully activated, allowing the SUFU/ GLI complex to enter the PC with the assistance of Kif7. Once at the tip of the PC, GLI dissociates from SUFU and remains in its full-length active form (GLIA). GLIA then translocates to the nucleus, where it drives the transcription of target genes.

Hh signaling is a phylogenetically conserved and elaborate intercellular communication mechanism. It has multiple key components and a complex modulatory network. Briefly, it primarily consists of four components, namely the ligand, receptor, signal transducer protein, and transcription factor, which can be categorized as being associated with canonical or noncanonical pathways depending on their signaling modes. The primary cilium (PC) is a non-motile, microtubule-based, highly specific organelle that protrudes from the cytoplasmic membrane and functions in a manner analogous to an antenna, capable of detecting extracellular signals [5]. The PC consists of a microtubule-based axoneme, basal body, and ciliary membrane. The microtubules of the axoneme are arranged in a “9 + 0” pattern, where nine doublet microtubules form a ring without a central pair. The basal body, which originates from the mother centriole, initiates and guides the assembly of cilia by providing a foundation for the axoneme’s extension. It is worth noting that not all cell types are ciliated [6]. Many components of Hh signaling are enriched in the PC. The maintenance of normal canonical signaling is strongly dependent on the PC [7], but noncanonical signaling can be achieved independent of the PC [8] (Figure 1).

### 2.1. Hh Ligand

Previous studies have confirmed that there are three types of Hh ligands in mammals: sonic hedgehog (Shh), Indian hedgehog (Ihh), and desert hedgehog (Dhh). Shh is widely present in multiple organs and tissue types, whereas Dhh is the most tissue-restricted, being mainly observed in the nervous system and testis [9,10,11,12]. Although these ligands exhibit differential expression patterns in terms of their levels and distribution, they exhibit high sequence homology and a shared signaling transduction mechanism [13]. The full-length Hh ligand protein precursor has a size of 45 kDa and consists of a signal peptide, an amino-terminal polypeptide (N-terminal), and a carboxy-terminal polypeptide (C-terminal) [14]. The N-terminal undergoes two lipid modifications—cholesterol and palmitate—via auto-processing and Hh acetyltransferase (Hhat), respectively [15,16]. During auto-processing, the cleaved C-terminal departs the endoplasmic reticulum and is degraded by the proteasome [13]. The mature Hh ligand, modified with dual lipids, can be liberated into the intercellular matrix through dispatched homolog 1 (DISP1), where its lipid-modified portion binds to the soluble carrier, signal sequence, cubulin (CUB) domain, and epidermal growth factor (EGF)-like protein 2 (SCUBE2) [17]. This SCUBE2-mediated soluble multimeric protein complex facilitates the natural long-range transport of hydrophobic, lipid-modified Hh ligands through the intercellular matrix [14]. Some researchers suggest that the active Hh ligand binds to the receptor patched (PTCH) via its lipid-modified domains, further altering PTCH’s function [18].

### 2.2. PTCH

The 12-pass transmembrane Hh signal receptor PTCH comprises two homologs, PTCH1 and PTCH2, with PTCH1 acting as the predominant receptor that is responsible for binding the Hh ligand in mammals [19]. PTCH1 regulates smoothened (SMO) activity via sterol transportation [14,20]. The Hh pathway is blocked by PTCH1, which inhibits SMO accumulation within the PC by reducing the sterol levels in the plasma membrane [21]. Upon Hh ligand coupling to PTCH1, there are co-receptors, including those cell adhesion molecule-related/downregulated by oncogenes (CDO), brother of CDO (BOC), and growth arrest-specific protein 1 (GAS1); these serve to enhance and stabilize Hh–PTCH1 complex formation [22]. The Hh–PTCH1 compound then undergoes ubiquitylation to be internalized and degraded [23]. On the other hand, the ligand can be sequestered by Hh interacting protein (HHIP), a negatively regulated co-receptor, to render the pathway silent [24].

### 2.3. SMO

SMO, a member of the class F G-protein-coupled receptor (GPCR) family, features ligand-binding sites: the seven-pass transmembrane (7TM) structural domain and the N-terminal extracellular cysteine-rich domain (CRD). Although the exact mechanism of SMO activation remains incompletely elaborated, growing biological and structural evidence suggests that its activation in mammals necessitates sterols that bind to the deep cavity within the 7TM domain, as well as the CRD site [25,26]. Moreover, the shallow cavity within the 7TM domain is the typical binding region for small-molecule SMO inhibitors (SMOis), such as cyclopamine, vismodegib, and sonidegib, where mutations can reduce drug affinity and lead to resistance [27,28,29]. In the absence of the Hh ligand, PTCH1, located on the PC, acts as a sterol transporter, making minimal sterols accessible to SMO, which results in SMO-inhibitory activity and rapid exit to the cytoplasm. Conversely, in the presence of the Hh ligand, PTCH1 allows SMO to enter the PC, where increased sterol activity within the ciliary membrane generates the optimal environment for SMO activation. Kinesin protein 3 (Kif3) and β-arrestin mediate the movement of SMO from the cytoplasm into the PC, where SMO activation occurs in strong association with the ciliary proteins EVC–EVC2 [30,31]. SMO must also be phosphorylated by both casein kinase 1 (CK1) and G-protein-coupled receptor kinase 2 (GRK2) to achieve the activated state [32].

### 2.4. GLI Family Transcription Factor

There are three known glioma-associated oncogene (GLI) family members in mammals: GLI1, GLI2, and GLI3. The DNA-binding capacity of GLI proteins is ascribed to their analogous zinc finger structural domains, which allow them to recognize and bind to the promoter regions of target genes, thereby regulating the transcriptional activity [33]. GLI2 is broadly utilized as a transcriptional activator, while GLI3 primarily acts as a repressor of transcriptional activity. *GLI1*, the GLI2 target gene, functions as a signal amplifier of the Hh pathway [34]. In the steady state, SMO is inhibited, allowing GLI, which is closely associated with suppressor of fused (SUFU), to be phosphorylated by several kinases, including protein kinase A (PKA), glycogen synthase kinase-3 (GSK3β), and CK1. This phosphorylation triggers ubiquitin-mediated proteolysis via β-transducin repeat-containing protein (βTRCP), converting GLI into the truncated version (GLIR), which is the repressor form [35,36,37,38,39]. GLIR then translocates to the nucleus, where it suppresses transcription. In the active state, SMO accumulates throughout the membrane of the PC. Kif7 aids in the movement of the GLI/SUFU complex through the PC [40]. Once at the tip of the PC, GLI dissociates from SUFU and retains its full-length activator form (GLIA), leading to nucleus translocation, the binding of DNA, and induction of the expression of Hh target genes, such as *vascular endothelial growth factor (VEGF)*, *snail*, α*-smooth muscle actin (*α*-SMA)*, and *desmin* [9,41].

### 2.5. Noncanonical Hh Signaling Pathway

In addition to the canonical Hh signaling pathway, including the Hh/PTCH1/SMO/GLI axis, three noncanonical Hh signaling pathways have been reported. Noncanonical Hh signaling of type 1 is PTCH1-dependent but independent of SMO and GLI. PTCH1 exerts proapoptotic and antiproliferative effects in the absence of the Hh ligand; it has been implicated in the activation of apoptosis-associated proteins such as caspase-3/9, DRAL, TUCAN-1, and cyclin B1. However, upon ligand binding to PTCH1, the noted effects are reduced [42,43]. Type 2 noncanonical Hh signaling is SMO-dependent but GLI-independent. This mode of SMO reflects its direct function as a GPCR through the activation of Gαi proteins; it activates protein kinases (Rho, Rac, and AMP-activated protein kinase (AMPK)) and calcium to regulate cytoskeletal arrangement and cell migration [44], which is related to Warburg-like metabolism in muscle and brown fat [45]. Finally, type 3 noncanonical Hh signaling refers to GLI-mediated transduction (Hh-GLI), irrespective of the upstream Hh ligand, PTCH, and SMO. Although the mechanisms of noncanonical Hh signaling have not yet been comprehensively elucidated, accumulating data indicate that multiple factors or pathways interact with GLI, either directly or indirectly. For example, it has been established that GLI2 is a direct downstream target of tumor growth factor-β (TGF-β) signaling [46]. GLI2 is reportedly activated by cJUN. Interestingly, GLI2 also enhances the expression of cJUN [47], which suggests that there is a positive feedback mechanism between them. Phosphatidylinositol 3-kinase (PI3K)/protein kinase B (AKT) signaling can not only activate but also repress GLI-mediated transcription [48].

Growing evidence suggests that both canonical and noncanonical mechanisms are collectively involved in disease onset and progression [49]. Notably, the inefficacy of drugs specifically designed to target the canonical pathway, such as SMOi, is caused, at least in part, by the noncanonical pathway. Therefore, understanding the relative contributions of these pathways to disease represents a significant challenge, but it is crucial for the improved characterization of disease mechanisms and the development of more effective targeted therapies.

## 3. Hh Signaling in Organ Fibrosis

The characteristics that are common to fibrosis across various tissue types and organs usually include the initial immunoinflammatory responses (a similar dynamic shift in macrophage phenotypes from M1 to M2), myofibroblast activation, tissue remodeling, and dissonant cell-to-cell signaling pathways [50]. Additionally, to capture this complexity, the term “fibrogenic niche” has been introduced and employed for diverse organs, such as the liver, lungs, and kidneys, demonstrating the fundamental similarity in fibrosis across these organs [51,52,53]. Chronic insults initially lead to a fibrogenic niche, subsequent fibrotic foci, and pervasive organ fibrosis, which eventually results in organ failure.

Myofibroblasts, which exhibit a contractile nature and express α-SMA, play a central role in fibrosis, as they are referred to as the most ECM-producing cells [54]. In healthy adults, the presence of myofibroblasts is nearly undetectable. Under normal physiological conditions, these cells typically undergo apoptosis (mostly), senescence, or inactivation once wound repair is completed and the inflammatory signals subside. Nevertheless, a dramatic increase in myofibroblasts, caused by the impaired resolution of these processes, leads to excessive ECM production and fibrotic tissue remodeling [55]. Regardless of the extensive research conducted so far, their precise cellular origins remain controversial. To date, substantial studies have shown that myofibroblasts can arise from various cell types, including resident fibroblasts, quiescent stellate cells, pericytes, bone-marrow-derived fibrocytes/mesenchymal stem cells (MSCs), endothelium through endothelial–mesenchymal transition (EndMT), epithelial cells via epithelial–mesenchymal transition (EMT), and GLI1^+^ MSCs. The contribution of each cell type to the myofibroblast population varies depending on the organ and the injury type [55,56]. Furthermore, single-cell transcriptomic, genomic, and proteomic studies have revealed the significant heterogeneity of these cells, highlighting their distinct regulatory functions within the fibrotic niche and the dynamic changes observed in fibrosis [57]. Given their indispensable role in fibrosis, the ability to selectively identify and target them, as well as reprogram them into cell types that preserve organ function, represents a promising strategy for antifibrotic therapies.

**Figure 2:**
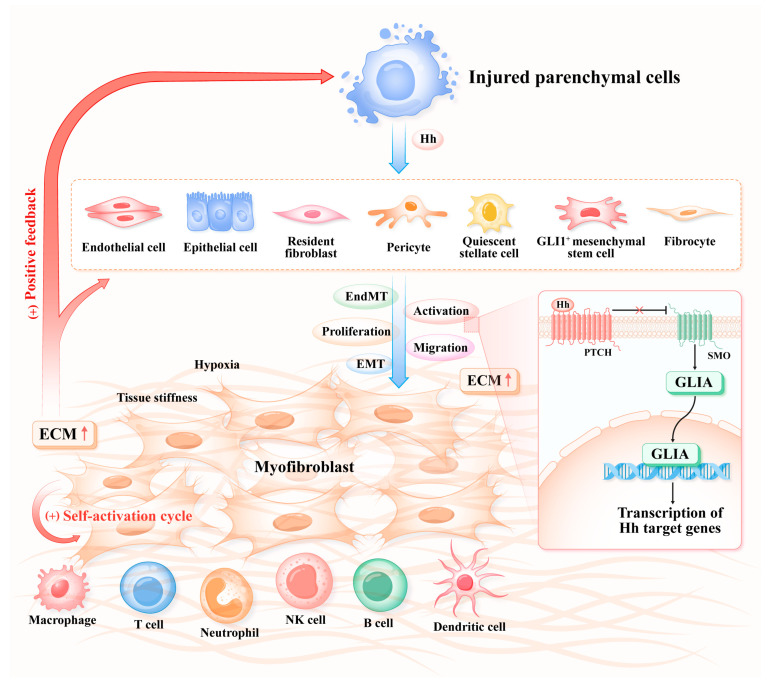
Hh signaling pathway mediated-fibrogenesis. Prolonged and repetitive stimuli lead to consistent injury of parenchymal cells and the recruitment of various inflammatory cells, including neutrophils, lymphocytes, macrophages, T cells, B cells, and dendritic cells. These cells secrete the Hh ligand in either an autocrine or paracrine manner. Several precursor cell types, such as resident fibroblasts, quiescent stellate cells, pericytes, bone marrow-derived fibrocytes/mesenchymal stem cells (MSCs), endothelial cells undergoing endothelial–mesenchymal transition (EndMT), epithelial cells undergoing epithelial–mesenchymal transition (EMT), and GLI1^+^ MSCs, which are responsive to the Hh ligand, contribute to the myofibroblasts’ activation, proliferation, differentiation, and sustained extracellular matrix (ECM) production. This process is accompanied by the activation of the Hh signaling pathway (Hh/PTCH/SMO/GLIA), ultimately resulting in increased tissue stiffness, hypoxia, and tissue remodeling, which together foster a vicious cycle.

Fibrosis is increasingly recognized as a highly dynamic and complex cellular and molecular process [58]. Parenchymal cells exhibit aberrant repair responses after chronic hits. Hh signaling facilitates cell-to-cell interactions among parenchymal cells, endothelial cells, mesenchymal cells, and inflammatory cells through mechanisms such as EMT, EndMT, and immune responses. These processes collectively contribute to the activation of collagen-producing myofibroblasts, the formation of an ECM network, and the progression of organ fibrosis. It also occurs in conjunction with other profibrotic mediators, including TGF-β, Wnt, Notch, and interleukin-4 (IL-4). These trigger a series of modifications, including tissue fibrotic remodeling, increased tissue stiffness, hypoxia, hypoperfusion, and metabolic alterations [50,59]. In turn, the fibrogenic niche creates a favorable environment for myofibroblast survival and proliferation, while impairing the regeneration and plasticity of the residual parenchymal cells, thus obstructing tissue repair. In advanced fibrosis, the self-reinforcing cycle within the microenvironment renders the reversal of fibrosis difficult but also accelerates the formation of cancer-associated fibroblasts and a tumor microenvironment [60]. This process underscores the critical role of the tissue microenvironment and multicellular interactions in the pathogenesis of fibrosis (Figure 2).

While they involve shared mechanisms, the tissue/organ fibrosis mediated by the Hh pathway also differs, particularly in terms of the target cell types and specific interaction mechanisms. These distinctions are critical for targeted therapies and clinical translation, warranting focused attention. In the following discussion, we explore the diverse roles of Hh signaling in organs such as the liver, kidneys, airways, and lungs.

### 3.1. Hh Signaling in Hepatic Fibrosis

Hepatic stellate cells (HSCs) are the primary source of hepatic myofibroblasts and serve as the key effector cells in liver fibrosis [61]. Besides HSCs, liver-resident mesenchymal cells, portal fibroblasts, and bone-marrow-derived cells such as fibrocytes and MSCs have also been identified as potential sources of hepatic myofibroblasts, depending on the etiology [55,62]. While hepatic epithelial cells, which constitute the majority of liver cells, do not directly contribute to the myofibroblast pool, their crosstalk with other cells—initiated by injury—plays a crucial role in driving the progression of liver fibrosis [63,64]. During fibrosis, injured hepatic epithelial cells undergo changes in their gene expression profiles and initiate a cascade of inflammatory events, releasing signals that recruit and activate macrophages, neutrophils, and lymphocytes to activate, proliferate, and mobilize HSCs [55].

Acute and chronic liver injuries lead to overexpressed Hh signaling in diverse cell types, with the Hh expression correlating with the severity and duration of the liver damage [23,65]. Firstly, Hh signaling acts in activated HSCs, with lipid droplet lossand the major ECM-producing cells, to regulate their expansion, metabolic reprogramming apoptosis, and senescence. Studies using murine models of hepatic fibrosis reveal that Hh-mediated metabolic reprogramming plays a critical role in fibrosis progression. Hh-driven increased glutamine catabolism is essential for HSCs to acquire and perpetuate a myofibroblastic phenotype. The α-ketoglutarate produced via glutaminolysis fuels the tricarboxylic acid cycle, providing the energy needed for activated HSCs [66,67]. Hh-Yes-associated protein (Hh-YAP) signaling has been shown to modulate this process, contributing to hepatic fibrosis, as demonstrated by the dramatically reduced myofibroblast-like HSC activity and mitochondrial metabolism when treated with cyclopamine and verteporfin (a YAP antagonist) [66]. Additionally, *hypoxia inducible factor-1* (HIF-1) is referred to as a GLI target gene. Hh signaling also determines the fate of HSCs via the upregulation of HIF-1α-induced glycolysis. A preclinical study has suggested that the conditional deletion of SMO specifically in HSCs reduces Hh signaling and significantly downregulates glycolytic and myofibroblast gene expression, whereas the pharmacological activation of Hh signaling with an SMO agonist, SAG, enhances their expression [68]. In addition, the role of Hh signaling in HSC senescence appears to be double-edged. Previous studies have emphasized that accelerating the senescence of activated HSCs can constrain and mitigate hepatic fibrosis, offering an effective strategy to treat fibrosis [69,70]. Nevertheless, emerging evidence reveals that senescent HSCs modify their secretory profiles, accompanied by the progressive accumulation of Hh signal molecules, such as Shh, which worsens the inflammatory and fibrotic microenvironment and accelerates the progression toward hepatocellular carcinoma (HCC) [71]. Secondly, previous dogma posited that the Hh ligand expression in healthy adult livers is minimal, suggesting a marginal role in regulating hepatic function under normal conditions. Interestingly, this concept has been challenged recently. Emerging evidence demonstrates that SMO defects in hepatocytes can promote metabolic derangement, fatty liver, and insulin resistance [72,73]. Another study reports that hepatocytes rapidly senesce when SMO is experimentally destroyed [74]. Chen et al. used human bulk single-cell RNA sequencing (scRNA-seq) data to show that SMO exhibited the strongest expression in healthy livers [73]. Thirdly, liver progenitor cells (LPCs) play a salutary role in liver regeneration following liver injury [75]. Recent work has identified EpCAM^+^ GLI1^+^ cells, a novel source of LPCs, as contributors to liver recovery [76]. On the other hand, Hu et al. found that theLPC-derived ductular reaction aggravates liver fibrosis through noncanonical Hh signaling. Interestingly, LPCs differentiate into hepatocytes via the downregulation of GLI1, which supports liver regeneration and alleviates cholestasis-induced fibrosis [77]. These findings suggest that the precise modulation of the Hh signaling pathway is essential for the full resumption of liver-specific function. Additionally, the capillarization of liver sinusoidal endothelial cells (LSECs) via the activation of Hh signaling impairs the maintenance of LSECs on HSCs’ quiescence [78]. Moreover, certain immune cells have been observed to exhibit responsiveness to Hh signaling. Upregulated Hh signaling has been linked to CD8^+^ T cell hyperfunction in chronic hepatitis C virus infection [79]. Osteopontin (OPN), an extracellular matrix glycophosphoprotein, is directly regulated by Hh signaling. In non-alcoholic steatohepatitis (NASH) mice, natural killer T cells exacerbated hepatic fibrosis via the Hh/OPN pathway [80]. Notably, the OPN silencing of GSK3β causes the enhanced transcription of GLI [81]. This mutual crosstalk suggests a positive feedback loop.

Collectively, these studies indicate that the Hh pathway plays distinct roles in different cell types, leading to the progression of hepatic fibrosis.

### 3.2. Hh Signaling in Renal Fibrosis

Renal fibrosis is a common and irreversible pathological characteristic of chronic kidney disease (CKD). Multiple studies regarding renal fibrosis have confirmed that resident fibroblasts, as well as pericytes, are the primary sources of myofibroblasts, with resident fibroblasts contributing more than pericytes [82]. Notably, EMT and EndMT have been reported to contribute negligibly as direct sources of fibrosis-producing cells [82]. Instead, certain soluble factors released by these cells into the mesenchyme may serve as drivers of fibrosis [58]. The formation of a fibrogenic niche accelerates the development of kidney fibrosis [52].

Extensive research has highlighted the correlation between renal fibrosis and amplified Hh signaling [83]. In the kidney tissue, the Hh ligands are expressed in tubule cells, and the interstitial cells express PTCH1, GLI1, and GLI2 [84]. An early mechanistic study proved that, in response to renal insults, tubule-cell-derived Shh facilitates the transformation of GLI1^+^ fibroblasts, ultimately leading to progressive fibrosis, which suggests that Hh signaling links kidney injury to fibrogenesis [85]. Nevertheless, another work provides a different perspective; using scRNA-seq, it identified a new inflammatory subset of proximal tubule cells as key players in renal fibrosis. This study found an increase in Ihh expression rather than Shh after injury. Tumor necrosis factor (TNF), produced by leukocytes in response to stimuli, activates the nuclear factor κB (NF-κB) pathway, driving Ihh release from a specific subset of ubiquitin D (Ubd)-expressing renal proximal tubular epithelial cells, resulting in kidney fibrosis. Elevated circulating Ihh has also been reported to be implicated in the diminished renal function in CKD patients. Encouragingly, kidney biopsies from CKD patients showed the presence of Ubd^+^ proximal tubule cells [86]. Moreover, this study revealed that the same outcomes were present in the aging kidney, emphasizing the key role of Ihh in both senescence and renal fibrosis progression [86]. Furthermore, the role of GLI1^+^ cells in organ fibrosis has been well established. Genetic fate-tracing experiments have indicated that GLI1-positive interstitial cells are a key source of kidney myofibroblasts [87]. The pharmacological blockade and genetic ablation of *GLI1* or *GLI2* in these MSCs ameliorated renal fibrosis [88]. In addition, researchers investigated the link between *GLI1* and *GLI2* mRNA expression and the extent of renal fibrosis in 10 human specimens, finding that increased *GLI1* and *GLI2* expression corresponded to a greater degree of fibrosis [89]. A core pathogenic mechanism in renal fibrosis is believed to be the crosstalk between injured tubular epithelial cells and mesenchymal cells, with extracellular vesicle (EV)-mediated communication playing a key role [90]. EV-encapsulated Shh, secreted from tubular epithelial cells, has been verified to favor fibrosis progression [91]. EVs not only facilitate the progression of disease through cell-to-cell communication within the kidneys but also offer a potential treatment for kidney fibrosis. Zhang et al. reported that human umbilical cord MSC-derived EVs improved kidney fibrosis in diabetic nephropathy via the inhibition of Hh signaling [92]. Notably, another study revealed the antifibrotic effect of a novel biological technology consisting of bioprinted MSC microfiber-derived EVs, which could enhance both the quantity and quality of EVs, in renal fibrosis [93], representing a promising avenue for potential clinical application in combination with biotechnology.

### 3.3. Hedgehog Signaling in Airway and Pulmonary Fibrosis

The fibrotic tissue response and remodeling are common characteristics of a spectrum of chronic pulmonary diseases, such as interstitial lung diseases (ILDs), asthma, and chronic obstructive pulmonary disease (COPD) [94]. The airway is covered by a layer of ciliated, secretory, and basal cells, which constitute an indispensable defensive barrier [95]. The distal alveolar epithelial cells consist of two distinct types: alveolar type 1 (AT1) and alveolar type 2 (AT2). AT2 cells possess the capacity to produce pulmonary surfactant, which is responsible for maintaining the alveolar surface tension and thereby averting alveolar collapse [96]. Surfactant protein C (SFTPC) serves as a specific marker for AT2 cells. In response to lung injury, AT2 can renew AT1 cells so as to repopulate damaged areas of the lung, thereby preserving the alveolar architecture and supporting lung tissue repair. The loss of AT2 cells, which are susceptible to injury, could be considered a precursor to fibrosis [96]. Growing evidence indicates that the molecular mechanisms leading to airway and pulmonary remodeling include the loss and metaplasia of epithelial cells, the recruitment of immune cells, EMT, EndMT, the transformation of fibroblasts to myofibroblasts, the excessive generation of ECM, and the formation of fibrous scars [97].

Hh signaling is associated with a range of chronic pulmonary diseases. Idiopathic pulmonary fibrosis (IPF) is the most frequent form of pulmonary fibrosis, with a poor prognosis. Histologically, IPF is especially characterized by “bronchiolization” and honeycomb cysts, accompanied by the ectopic appearance of cytokeratin 5 (KRT5*^+^*) basal cells in the distal lung and fibrous scarring, which impairs respiratory function [98]. The disrupted distal alveolar niche and a decline in SFTPC^+^AT2 cells have been demonstrated to result from the peculiar expansion of Hh signaling in the alveolar mesenchyme [99]. GLI1*^+^* MSCs have been observed to migrate into the alveolar region, thereby establishing a pathological niche in association with Hh signaling activation. The underlying mechanism involves accelerating the metaplastic differentiation of airway progenitors into KRT5*^+^* basal cells and promoting fibrotic scar formation [100]. It has been reported that Hh pathway expansion in GLI1*^+^* MSCs instigates the progression of bleomycin-treated pulmonary fibrosis by upregulating bone morphogenetic protein antagonism [100]. Moreover, the dysregulated activation of Hh signaling is increasingly being recognized as a significant contributor to airway remodeling and the progression of chronic inflammation in COPD and asthma. Genome-wide association studies of human chronic airway diseases have implicated the *HHIP* locus as a key susceptibility factor for COPD, with *PTCH1* identified as a risk locus for both COPD and asthma [101,102]. Both HHIP and PTCH1 function as central negative feedback regulators within the Hh pathway. In an in vitro study using a cigarette-induced COPD model, it was found that cyclopamine could attenuate airway inflammation to varying degrees [103]. *HHIP^+/−^* mice exhibited a greater extent of and susceptibility to emphysema, which have been utilized in COPD research [104]. The investigations by Yun et al. in *HHIP^+/-^* mice reveal that HHIP plays a critical role in suppressing inflammatory lymphocyte infiltration into the lungs. Specifically, reduced HHIP expression correlates with increased IL-18 derived from lung fibroblasts, which facilitates CD8^+^ T cell activation and type I interferon (IFN)-γ production [104].

Evidence indicates that the Hh signaling pathway plays a non-negligible role in intercellular crosstalk via the regulation of the immune–inflammatory responses. The M1/M2 types have always been an important framework for macrophage studies in pulmonary fibrosis [105]. The proinflammatory cytokines generated by M1 macrophages, such as TNF-α and IL-1β, have been proven to be tightly interlinked with ILD [105]. In a model of silica-induced lung fibrosis, the prolonged activation of the NOD-like receptor protein 3 (NLRP3) inflammasome via the uncontrolled Shh/GLI and Wnt/β-catenin pathways facilitated the inflammatory microenvironment, including neutrophil, lymphocyte, and monocyte infiltration; the increased production of TNF-α and IL-1β; and enhanced EMT in the distal region [106]. Hou et al. found that Shh-producing AT2 cells boosted macrophage M2 polarization and bleomycin-induced pulmonary fibrosis progression via the Shh/GLI1/OPN/Janus kinase (JAK) 2/signal transducer and activator of transcription (STAT) 3 cascade [107]. The silencing of Shh signaling also markedly decreased the levels of neutrophils and lymphocytes in bronchoalveolar lavage (BAL) fluid [107]. Additionally, studies have shown the involvement of multiple T cell subsets, including T helper lymphocyte (Th)1, Th2, and Th17 and regulatory T cells (Tregs), in the airway and in pulmonary fibrosis. Experimental models of lung fibrosis have identified the antifibrotic properties of Th1 cells and the pathogenic effects of Th2 cells [108,109]. However, the exact mechanism behind them has not yet been completely clarified [96]. Notably, the central role of Hh signaling in the management of T cell differentiation has been established in mice and human subjects. In vitro research found that Shh signaling drives human Th2 differentiation, with the concomitant suppression of IFN-γ and T-bet, which appears to be implicated in the GLI2-targeted upregulation of the profibrotic Th2 cytokine IL-4 [110]. In concordance with this, Shh-driven Th2 differentiation in allergic airway disease has been widely discussed [111]. Using a papain-induced mouse model of allergic airway disease, Yanez et al. demonstrated that systemic pharmacological SMO inhibition significantly attenuated Th2 inflammation in the lungs, including the lowering of T cell, eosinophil, basophil, and mast cell infiltration and a decline in Shh, IL-4, and IL-13 expression, as well as the serum immunoglobulin E concentrations [112]. Notably, Jin et al. reported that Hh signaling was also essential for the Th17 response in a model of asthma that was insensitive to corticosteroids. After treatment with cyclopamine, the polarization of Th17 and Th2 was decreased, whereas Treg and Th1 polarization was enhanced [113]. IL-17A, which is a Th17 cytokine, has been suggested to act in lung fibrotic remodeling and facilitate EMT and fibroblast recruitment and activation [114]. In conclusion, these studies demonstrate the Hh signaling as a promising potential therapeutic target for chronic pulmonary diseases to alleviate airway and lung tissue remodeling.

Hh signaling can also modulate airway and lung fibrosis by noncanonical pathways. GANT58, a GLI1 inhibitor, has been indicated to downregulate the *Wnt* genes (*Wnt7b* and *Wnt10a*). Subsequent results reveal that the possible crosstalk mechanism involving TGF-β1/GLI1/Wnt/β-catenin signaling drives pulmonary fibrosis progression [115]. Additionally, an in vitro asthma study indicated that cyclopamine and GANT61 alleviated the TGFβ1-induced increase in *COL1A1* expression, implying that the activation of Hh signaling in asthma is caused, at least in part, by TGF-β, resulting in the airway remodeling [116]. Lately, independent crosstalk mechanisms between HHIP and TGF-β are associated with enhanced EMT in human bronchial epithelial cells, contributing to the pathogenesis of COPD [117].

### 3.4. Hedgehog Signaling in Other Fibrosis

Abnormal Hh pathway activation has been linked to a multitude of human fibrotic diseases, including cardiac fibrosis [41,87,118,119], skin fibrosis [120,121], systemic sclerosis (SSc) [47,122], myelofibrosis (MF) [123,124,125,126], arterial fibrosis [127], cystic fibrosis [128], chronic pancreatitis [129,130], inflammatory bowel disease (IBD) [49,131], peritoneal fibrosis [132], benign prostatic hyperplasia [133], endometrial fibrosis [134], oral submucous fibrosis [135], ligamentum flavum fibrosis [136], and arthrofibrosis [137].

Hh signaling exhibits a dual role in cardiac repair and fibrosis. The literature on cardiac Hh signaling has primarily focused on its role in angiogenesis and its protective effects on cardiomyocytes. An early study suggested that Shh signaling acts in acute or chronic myocardial ischemia to ameliorate left ventricular fibrosis, protect myocardial cells by alleviating apoptosis, and enhance neovascularization, accompanied by the upregulated expression of VEGF [41]. On the other hand, a lineage tracing investigation indicated that resident perivascular Gli1^+^ MSCs contribute to the myofibroblast pool following heart injury [87]. The genetic ablation of *GLI1* attenuated cardiac fibrosis and salvaged the ejection fraction in a cardiac fibrosis model [87]. Another study demonstrated that intact fibroblast growth factor 23 could trigger fibroblast expansion and transformation into myofibroblasts, promoting myocardial fibrosis through Shh signaling [119]. These findings suggest that Hh signaling may play different roles depending on the cell type, timing, and microenvironment [86].

Moreover, in experiments, the pharmacological inhibition and genetic deletion of *GLI1* in MSCs have been found to alleviate bone marrow fibrosis [125]. A recent study revealed that JAK2/STAT3 directly promoted *GLI1* transcriptional activity in fibrocytes rather than MSCs, thereby contributing to MF [123]. Ruxolitinib, a JAK2 inhibitor, has been used in the clinical treatment of MF patients. Preclinical studies have indicated that the combination of ruxolitinib with sonidegib results in greater reductions in bone marrow fibrosis, JAK2 mutant allele burden, and platelet counts compared to ruxolitinib used alone [138].

Hh signaling exerts intricate effects on the immune microenvironment in intestinal inflammation and fibrogenesis. In an animal model of colitis, Shh/GLI caused the robust upregulation of IL-10 and Treg recruitment to quell inflammation [139]. On the other hand, the overexpression of Hh signaling is pathogenic. Hanna et al. found that endogenous Ihh can drive Th17 polarization via both canonical and noncanonical (SMO/AMPK) pathways, which are implicated in IBD. Vismodegib has been proven to greatly attenuate inflammation in the murine intestine [49].

Abnormal Hh signaling has also been associated with skin-related pathologies. In the endothelium, it was found that the conditional genetic deletion of PTCH1 led to heightened EndMT and excessive skin scarring [121]. Of particular note is the fact that Hh signaling may be favorable in chronic inflammatory conditions of the skin, which is in stark contrast to its effects in the lungs [140]. The pharmacological suppression of SMO exacerbated skin inflammation in a rodent model of atopic dermatitis (AD). Specifically, Shh signaling favors Treg accumulation and activation via TGF-β, with a striking decrease in Th1-, Th2-, and Th17-related cytokines, thus tending to provide an immunosuppressive environment in the skin [141].

In conclusion, as mentioned above, Hh signaling exerts distinct effects across various organs. For example, in the liver, the abnormal upregulation of Hh signaling drives HSC activation, which is central to Hh-driven liver fibrosis, while the depletion of Hh signaling in hepatocytes is more closely associated with lipid accumulation, metabolic dysregulation, aging, and NASH progression. In the heart, Hh activation in the early stages of injury primarily aids cardiomyocyte repair (beneficial activation), while, in the sustained stages, it contributes more to profibrotic effects in fibroblasts (adverse activation). The Hh-mediated EMT is a significant mechanism in airway and lung fibrosis, but it contributes minimally to kidney fibrosis. The inhibition of Shh signaling exacerbates chronic skin inflammation (e.g., in AD, as Hh drives Treg differentiation), whereas the opposite effect is observed in the airways and lungs, where it alleviates chronic inflammation and tissue remodeling (e.g., in asthma, where Hh drives Th2 and Th17 differentiation). Despite the precise mechanisms underlying these differences remaining incompletely understood, these observations all highlight the context dependence and tissue specificity of Hh signaling. As a morphogen, the Hh protein’s intensity and duration determine distinct cell fate patterns, implying that, depending on the disease stage and the presence of specific Hh-responsive cell populations, Hh signaling may exhibit diverse roles and varying contributions to the pathophysiology of fibrosis across different tissues and organs. This may explain the differing outcomes of Hh-mediated fibrosis across organs. Future studies are needed to further elucidate the exact mechanisms by which the time, environment, and cell type influence Hh signaling, which will be crucial in determining how to precisely modulate Hh signaling to achieve personalized therapeutic targets.

## 4. Therapeutic Implications

Despite huge progress achieved in recent years, at present, the pharmaceutical options available for the clinical management of organ fibrosis remain limited, with pirfenidone and nintedanib being the only antifibrotic agents approved for IPF by the Food and Drug Administration (FDA) [97,142]. The recognition of the pivotal role of Hh signaling in a diverse array of organ fibrosis has aroused considerable interest in targeting this pathway for antifibrotic treatment. Advances in our understanding of Hh signaling, combined with high-throughput sequencing technology, have driven progress in the development of Hh-targeted drugs over the past few decades. These drugs can be categorized into small-molecule inhibitors, antibodies, natural compounds, and oligonucleotides [13], with small-molecule inhibitors being the most prominent. They are capable of impeding various components of Hh signal transduction, such as Hhat, Shh, SMO, and GLI inhibitors. Indeed, most Hh-related drugs that are currently undergoing clinical trials were originally designed to treat cancers such as basal cell carcinoma, HCC, and acute promyelocytic leukemia [44,143,144], rather than organ fibrosis. As previously described, cumulative data obtained in vivo and in vitro have yielded insights into their significant antifibrotic potential. Unfortunately, many of these drugs remain in the preclinical stages, with only some advancing to clinical trials (Table 1 and Table S1). In this review, we provide an updated summary of the therapeutic strategies targeting Hh-driven fibrosis and discuss their antifibrotic potential and clinical applications (Figure S1).

### 4.1. SMO Inhibitors

Among these, SMOis based on cyclopamine, a plant-derived steroidal alkaloid, have yielded the most notable success, gaining approval from the FDA for the treatment of cancer. They include vismodegib, sonidegib, and glasdegib [145,146,147]. Moreover, other SMOi drugs, including taladegib, itraconazole, and saridegib have been subjected to clinical trials.

#### 4.1.1. Cyclopamine

Cyclopamine was the first naturally occurring SMO antagonist to be identified, with its discovery initially linked to developmental malformation in lambs. Subsequently, it has been extensively employed in the investigation of the Hh pathway [148]. Both in vivo and in vitro studies have verified its satisfactory antifibrotic efficacy [66,149,150]. Unfortunately, its poor pharmacokinetics, lack of specificity, and toxicity have precluded its progression to clinical trials [151].

#### 4.1.2. Vismodegib (GDC-0449)

Vismodegib, which is a second-generation cyclopamine derivative, has been linked to several liver-related adverse events in clinical studies, including hepatocellular injury, hepatitis, ascites, hepatotoxicity, and acute liver failure [152,153]. In one clinical study comprising 15 patients treated with vismodegib, two patients exhibited a two-fold increase in alkaline phosphatase levels [153]. Accordingly, further validation is required to ascertain the secure dosage for clinical application. A phase 1b, open-label, multicenter trial on vismodegib in combination with ruxolitinib, conducted on 10 subjects with MF, evaluated the safety and efficacy of this combination. Although the treatment was generally well tolerated, this study was terminated due to the limited additional improvement compared to a ruxolitinib monotherapy [154]. Furthermore, in a murine model of bleomycin-induced pulmonary fibrosis, vismodegib showed no effect on lung fibrosis [155]. In alignment with this, a phase 1b, open-label trial consisting of 21 patients with IPF who were treated with a combination of vismodegib with pirfenidone did not yield any conclusive results and was terminated due to safety concerns [156].

#### 4.1.3. Sonidegib (LDE225)

A lower incidence of overall adverse events has been observed in patients treated with sonidegib as compared to vismodegib. A phase 1 clinical trial demonstrated that sonidegib (800 mg orally administered) was well tolerated in subjects with severe hepatic impairments [157]. Furthermore, the effect of sonidegib plus ruxolitinib in MF patients was evaluated in a phase 1b/2 study; they received 400 mg sonidegib daily and 20 mg ruxolitinib twice daily for 24 and 48 weeks. During this study, the spleen volume was reduced by at least 35% in 44.4% and 29.6% of the MF patients, respectively. However, this study was ultimately halted due to a lack of added benefit compared to a ruxolitinib monotherapy [158]. Another phase 1 trial in 17 patients with steroid-refractory chronic graft-versus-host disease (cGVHD) found that sonidegib decreased the Hh-related protein expression, as evidenced by an immunohistochemical analysis of skin biopsies. During the trial, 47% of the patients had a partial response, while 35% of the patients exhibited no response. Unfortunately, this study was also terminated due to safety concerns and poor compliance [159].

#### 4.1.4. Saridegib (IPI-926)

Saridegib, a semisynthetic cyclopamine, markedly enhances the pharmaceutical potency and has a superior pharmacokinetic profile compared to cyclopamine [160]. Experimentally, saridegib was found to be ineffective in diminishing kidney fibrosis [84], a finding that was mirrored in a clinical study. Saridegib alone has been evaluated in MF with 14 patients. During this study, 12 patients exhibited minor reductions in their spleen volume, yet no discernible alleviation of symptoms was observed, with the most common side effects being gastrointestinal and hepatic abnormalities [161]. Thus, in contrast to other types of SMOi, saridegib might be a less optimal candidate for the treatment of organ fibrosis, necessitating further preclinical and clinical studies to assess its efficacy in future applications.

#### 4.1.5. Glasdegib (PF-04449913)

Glasdegib exerts antifibrotic effects by acting as a small-molecule inhibitor that binds to SMO. Two clinical studies have found that glasdegib displays acceptable safety in subjects with hepatic and renal impairments [162,163]. Moreover, glasdegib displayed promising responsiveness in two phase 1/2 studies in patients with refractory sclerotic cGVHD. However, muscle cramping emerged as the most frequently reported adverse effect, which severely compromised its tolerability and limited its broader clinical application [120].

#### 4.1.6. Taladegib

The clinical development of taladegib for the treatment of IPF is progressing rapidly, with the drug currently undergoing a phase 2 trial (NCT06422884). It is anticipated to provide a new option for IPF.

#### 4.1.7. Itraconazole

Itraconazole, which has been approved by the FDA as an antifungal agent, has also been demonstrated to enable the blockade of the Hh pathway by hindering SMO accumulation within PC [164]. It has demonstrated good safety and tolerability in clinical settings [165]. In vivo and in vitro studies have indicated that itraconazole can effectively ameliorate liver fibrosis [166].

Although preclinical data have shown the significant efficacy of SMOis in reducing tissue and organ fibrosis, the therapeutic outcomes and safety in clinical treatments have been less than satisfactory. In addition to limitations in model translation, SMOi resistance—primarily due to *SMO* mutations and the activation of noncanonical pathways—should not be neglected. Indeed, emerging evidence suggests that noncanonical pathways, particularly Hh-GLI, play a crucial role in fibrotic diseases. Several known profibrotic factors and pathways, such as TGF-β, VEGF, EGF, PI3K/AKT, and JAK/STAT, have been reported to positively regulate GLI activity independent of the canonical pathway [48,167,168,169,170]. Preclinical data illustrated that SMOis failed to reverse pulmonary fibrosis, kidney fibrosis, and MF, whereas the direct inhibition of the GLI protein by GANT61 abated fibrosis in these tissues [84,125,155]. In summary, the application of SMOis in patients with fibrosis is challenging due to the complicated interplay among profibrotic pathways and the diverse cellular mechanisms involved in fibrosis. Furthermore, the adverse effects observed in treated patients, including ageusia, nausea, diarrhea, and hair loss, present barriers to clinical application. Therefore, it is necessary to develop downstream-targeted drugs, reasonable multi-targeted drugs, and nanoparticle (NP)-based drug delivery systems together to improve the clinical potential of Hh-targeted therapies. Moreover, given that the current clinical trials for fibrosis that involve SMO antagonists are less plentiful and small, larger clinical studies are required to comprehend their efficacy and safety.

### 4.2. GLI Inhibitors

As mentioned previously, owing to its location downstream of Hh signaling and at the crossroads of various profibrotic pathways, such as TGF-β and Wnt, the transcription factor GLI could theoretically overcome SMOi resistance and become a more efficacious target for pharmacological inhibition.

#### 4.2.1. GANT58 and GANT61

GANT58 and GANT61 are currently the most broadly deployed small-molecule GLI blockers; they are attached to DNA to downregulate GLI1/2 transactivation. Extensive experimental research has suggested that GANT compounds act as Hh inhibitors to effectively suppress the activation, expansion, and migration of myofibroblasts, thereby alleviating fibrosis, including that in the liver, lungs, kidneys, heart, and skin. However, these promising results have not been replicated in vivo as a consequence of their poor solubility, rapid clearance, and low bioavailability [171]. At present, there is a striking paucity of clinical evidence about GANTs.

#### 4.2.2. Darinaparsin (ZIO-101)

Darinaparsin is an organic arsenic trioxide derivative that has been approved in Japan for the treatment of relapsed or refractory peripheral T cell lymphoma [172]. Research has shown that darinaparsin inhibits Hh signaling by downregulating GLI2 transcription [173,174]. Kramann et al. demonstrated that GLI2 could be inhibited by darinaparsin, since it is referred as to a direct target of darinaparsin, which contributes to cell cycle arrest in myofibroblasts and renal fibrosis regression in a mouse model [88].

In summary, while inhibiting the Hh pathway at the GLI transcriptional level may offer a more stable antifibrotic effect compared to targeting SMO, and encouraging results have been found in some preclinical animal studies, subsequent clinical trials on GLI-targeted drugs, such as GANT compounds, appear to be frustrating due to their cytotoxicity, instability, lack of selectivity, and severe side effects, which hinder their clinical use. Furthermore, the potential off-target effects associated with targeting GLI should not be overlooked.

### 4.3. Other Drugs with Potential Associations with the Hh Signaling Pathway

#### 4.3.1. Pirfenidone

Although the precise mechanism of pirfenidone is not fully understood, it has been established that pirfenidone can interfere with TGF-β to exert certain therapeutic effects [175]. Additionally, a study revealed that pirfenidone could inactivate the primary lung fibroblasts in IPF patients, concomitant with a reduction in α-SMA and fibronectin expression via the blocking of GLI transcription [176]. Another recent study on pirfenidone also reported its antifibrotic effect through Hh-mediated EndMT reduction [177]. In a small study on 25 patients with SSc-ILD, pirfenidone was reported to attenuate lung fibrosis via strengthening Hh repression through SUFU and GSK3β [178].

#### 4.3.2. Empagliflozin

Empagliflozin, a sodium–glucose cotransporter-2 inhibitor, is a well-established hypoglycemic drug that is approved for the treatment of type 2 diabetes mellitus. A preclinical study has demonstrated that empagliflozin exerts antifibrotic effects through the suppression of the Hh pathway, leading to reduced inflammation, HSC activation, ECM deposition, and liver fibrosis in a murine model [179].

#### 4.3.3. CX-4945 (Silmitasertib)

CK2, a serine/threonine kinase and a driver of Hh signaling, has been reported to be related to SSc [180]. The CK2 inhibitor CX-4945 is currently being used in clinical trials for the treatment of locally advanced/metastatic cholangiocarcinoma, along with gemcitabine and cisplatin [181]. Fan et al. suggest that the experimental administration of CX-4945 downregulates the CK2/SMO pathway and partially reverses fibrosis and HSC activation [182].

### 4.4. NP-Based Drug Delivery Systems

The sustained, deleterious crosstalk among various cellular components within the fibrogenic niche drives the onset and progression of fibrosis. Therefore, the single-agent approach targeting the Hh pathway has achieved only modest efficacy. Moreover, conventional drug therapies frequently encounter constraints, including their inherent toxicity, weak specificity, suboptimal bioavailability, and comparatively short half-lives [183]. As precision medicine has advanced, targeted therapies have emerged as the most promising solutions. In recent years, NP-based drug delivery systems have emerged as a promising avenue to surmount the obstacles posed by otherwise prospective drug candidates and exhibit tremendous potential. A study by Zhang et al. indicated that an HSC-targeted versatile NP delivery system relying on the attachment of targeting moieties, hyaluronic acid, and cRGDyK peptide effectively abrogated activated HSCs and alleviated liver fibrosis in mouse models, without detectable toxicity. This was achieved through a combination of Hh pathway inhibition and the activation of the nuclear factor erythroid 2-related factor 2 signaling pathway [184]. Moreover, Zhang et al. designed two lipid NP (LNP) delivery systems, which were modified with chondroitin sulfate and glycyrrhetinic acid and loaded with vismodegib and silybin. This combination not only facilitated the restoration of capillarized LSECs and deactivated HSCs by blocking Hh signaling, but also attenuated hepatocyte damage via ROS reduction [185]. In contrast to most drug delivery systems, which rely on targeting moieties, Younis et al. explored ligand-free, siRNA-entrapped LNPs, which significantly inhibited HSC activity and halted the progression of liver fibrosis by simultaneously silencing the Hh and TGF-β signaling pathways [186]. In summary, these findings suggest that this is a promising therapeutic candidate for hepatic fibrosis.

Certain natural compounds and non-coding RNAs have been demonstrated to exert a pivotal influence on fibrosis through interactions with Hh signaling [187,188,189,190]. Furthermore, circulating Ihh may be considered a reliable biomarker of an adverse clinical prognosis, as it is negatively associated with organ function, including that of the kidneys, liver, and heart [86,191]. These findings collectively underscore the necessity to comprehensively understand Hh signaling to devise optimized therapeutic interventions and to create new biomarkers that can assist in the diagnosis and monitoring of treatment progress.

## 5. Conclusions and Future Clinical Perspectives

Despite the considerable progress achieved in understanding the mechanisms of Hh and its role in fibrosis, it must be noted that bottlenecks and challenges remain in the translation of this knowledge from the bench to bedside. Firstly, as discussed earlier, although there is substantial evidence linking Hh signaling with organ fibrosis and demonstrating the promising antifibrotic effects of its targeted inhibition—mostly informed by preclinical models—clinical trials often fail to reach their endpoints and achieve satisfactory therapeutic outcomes. Presumably, these limitations partially arise from the context-dependent nature of Hh signaling and the abnormal activation of its noncanonical pathway. Moving forward, it will be essential to elucidate the extent to which Hh signaling contributes to fibrosis across various organs, including an assessment of the relative contributions of canonical and noncanonical pathway involvement, which may aid in identifying more strategies to precisely modulate the Hh pathway and in developing individualized interventions for fibrosis patients. Secondly, as a highly conserved developmental pathway, the important role of Hh signaling in adult tissue repair has been established, including in the heart and liver. Additionally, the loss of GLI1^+^ cells in the kidneys has been associated with capillary rarefaction, in turn inducing renal fibrosis [192]. Similarly, the ablation of SMO in the entire pancreatic epithelium or specifically in adult acinar cells severely impairs the physiological exocrine function of the pancreas [193]. Therefore, the disruption of this pathway could lead to physiological imbalances, raising the question of whether inhibiting Hh signaling is safe for fibrotic patients; it is critical to examine this aspect, and it should be managed with caution. Thirdly, growing evidence suggests that the noncanonical pathway plays a significant role in fibrosis; this means that multiple signaling cascades may work in concert to promote fibrosis, increasing the difficulty and complexity of the corresponding therapies. Lastly, a single hit may be insufficient to arrest fibrotic progression. Multiple pathways that drive ECM-producing processes need to be restrained in tandem to prevent the complex crosstalk among uncontrolled parenchymal cells, inflammatory cells, and fibrosis-secreting cells. Hence, NP-based drug delivery systems, multi-targeted therapies, and other combinational strategies might be necessary to disrupt the vicious circle within the fibrotic microenvironment and reduce the likelihood of developing drug resistance. Importantly, long-term results need to be assessed before any definitive conclusion can be drawn.

Overall, the impact of Hh signaling on fibrotic diseases has been confirmed, and there are ongoing efforts to inhibit Hh-driven fibrosis in clinical therapies. Thus, we emphasize that Hh signaling remains a promising target for therapeutic intervention in fibrosis.

## Figures and Tables

**Table 1:** Summary of anti-fibrotic drugs in clinical trials targeting Hh signaling.

Drugs	Target Protein	NCT	Status	Disease	Study Completion	Study Design/Sample Size, Planned or Enrolled	Dosage Regimen	Duration	Primary Endpoint	Outcome
Vismodegib	SMO	NCT02593760	Completed	MF	2017-07	Phase 1b, multicenter, randomized, double-blind, placebo-controlled trial, *n* = 10	Vismodegib (150 mg orally once daily) + ruxolitinib (15/20 mg orally twice daily, depending on baseline platelet count)	24–48 weeks	(1) Percentage of participants with ≥35% reduction in spleen volume from baseline at week 24 (2) Percentage of participants with CR and PR at week 24	No significant difference between groups
		NCT02648048	Completed	IPF	2016-11	Phase 1b, single arm, multicenter, open-label trial, *n* = 21	Vismodegib (150 mg orally once daily) + pirfenidone (≤108 mg orally three times daily)	24–48 weeks	Safety and tolerability	No significant difference between groups
Sonidegib	SMO	EudraCT No. 2011-005876-40	Completed	IHF	No information	Phase 1, parallel group, multicenter, open-label trial, *n* = 33	Sonidegib (800 mg orally once daily)	11 weeks	Compare the PK of a single oral dose of sonidegib in IHF subjects and healthy subjects	No significant difference between groups
		NCT01787552	Completed	MF	2018-04	Phase 1b/2, open-label, multicenter, dose-finding trial, *n* = 15	Phase 1b: sonidegib (400 mg daily) + ruxolitinib (10/15/20 mg twice daily) Phase 2: sonidegib (400 mg daily) + ruxolitinib (20 mg twice daily)	24–48 weeks	Phase 1b: incidence of DLTs in the first 6 weeks Phase 2: spleen volume reduction	No significant difference between groups
		NCT02151864	Completed	Cirrhosis	2017-09	Phase 1, open-label, single institution, dose escalation trial, *n* = 9	Sonidegib (200–800 mg orally daily)	42 days	Rate of DLT	No information
		NCT02086513	Terminated	cGVHD	2016-12	Phase 1 trial, *n* = 17	Sonidegib (200/400/600 mg orally once daily in 28-day cycles)	12 cycles of 28 days each	Incidence of DLTs in the 2 cycles	Terminated due to safety concerns and poor compliance
Saridegib	SMO	NCT01371617	Completed	MF	2012-08	Phase 2, single-arm trial, *n* = 14	Saridegib (160 mg orally once daily in 28-day cycles)	7.5 months	Overall response rate: clinical improvement, PR, and CR	No significant difference between groups
Glasdegib	SMO	NCT03596567	Completed	Renal impairment	2018-09	Parallel-group, open-label trial, *n* = 18	Following an overnight fast of ≥ 10 h, glasdegib (100 mg orally once daily)	35 days	The effect of renal impairment on the PK	No significant difference between groups
		NCT03627754	Completed	IHF	2019-04	Phase 1, open-label, parallel group, single dose trial, *n* = 24	Following an overnight fast of ≥ 10 h, glasdegib (100 mg orally once daily)	35 days	Total plasma glasdegib AUCinf and the Cmax of glasdegib	No significant difference between groups
		NCT03415867	Unknown status	cGVHD	2022-06	Phase 1b/2a, Open label, multi-center trial, *n* = 20	Phase 1b: glasdegib(50 mg once daily in 28-day cycles) Phase 2b: glasdegib(25/100/150/200 mg)	12 months	DLT and MTD	Response rates of 65% in skin/joint scGVHD after treatment
		NCT04111497	Terminated	cGVHD	2023-08	Phase 1b/2a, open label, multi-center trial, *n* = 15	Glasdegib (50 mg, adjusted according to treatment-related toxicity grade)	12 months	Safety and tolerability (type and severity of AE, time on treatment, and reasons for discontinuation)	Response rates of 47% in skin/joint scGVHD after treatment
		NCT02226172	Terminated	MF	2018-01	Phase 2, double-blind, randomized, placebo-controlled trial, *n* = 21	Glasdegib (100 mg orally once daily in 28-day cycles)	24–131 weeks	(1) Percentage of participants with ≥ 35% reduction in spleen volume from baseline at week 24 (2) Number of participants with treatment AEs and serious AEs (3) Number of participants with laboratory abnormalities	No information
Taladegib	SMO	NCT05817240	Completed	IPF	2023-06	Phase 1, fixed sequence, drug-drug Interaction trial, *n* = 21	Taladegib (100 mg once daily) + nintedanib (100 mg once daily)	7 days	Nintedanib Cmax, Tmax, AUC0-t, AUC0-inf, T1/2 administration alone and after coadministration with taladegib	No information
		NCT04968574	Completed	IPF	2023-11	Phase 2, randomized, placebo-controlled, multicenter trial, *n* = 41	Taladegib (200 mg once daily for 12 weeks)	18 weeks	Change from baseline in frequency of AEs; severity of AEs; pulse; blood pressure; respiration rate; temperature; blood oxygen saturation level; incidence of clinical laboratory abnormalities; severity of clinical laboratory abnormalities; and number of hospitalizations	No information
		NCT06422884	Ongoing	IPF, PPF	2026-06	Phase 2, randomized, placebo-controlled, double-blind, dose-ranging trial, *n* = 320	Taladegib (orally once daily)	6 months	(1) IPF: Rate of change in percent ppFVC (2) PPF: The incidence, severity, and relationship of AEs. Changes in lab parameters, vital signs, electrocardiogram, and oxygen saturation	Results awaited

Abbreviations: IPF: idiopathic pulmonary fibrosis; CF: cystic fibrosis; MF: myelofibrosis; CR: complete remission; PR: partial remission; cGVHD: chronic graft-versus-host disease; IHF: impaired hepatic function; PPF: progressive pulmonary fibrosis; NASH: non-alcoholic steatohepatitis; IBD: inflammatory bowel disease; AE: adverse events; MTD: maximum tolerated dose; PK: pharmacokinetics; DLT: dose-limiting toxicity; IHF: impaired hepatic function; Cmax: maximum blood concentration; Tmax: Time of drugs maximum blood concentration; AUC0-t: absorption to time t; AUC0-inf: total absorption; T1/2: half-life in the blood; ppFVC: percent predicted forced vital capacity.

## Data Availability

Not applicable.

## Supplementary Materials

The following supporting information can be downloaded at: https://www.mdpi.com/article/10.3390/biom14121485/s1, Figure S1: Chemical structures of anti-fibrotic drugs related to the Hh signaling pathway discussed in the review; Table S1: Clinical trials of anti-fibrotic drugs associated with the Hh pathway discussed in this review.
