# Supplementary material for: Hedgehog Signaling Pathway in Fibrosis and Targeted Therapies
Source: Biomolecules. 2024 Nov 22;14(12):1485. doi: 10.3390/biom14121485 (PMC11727624; doi:10.3390/biom14121485)
Supplement: Supplementary file 1 [file biomolecules-14-01485-s001.zip › biomolecules-3302938-supplementary.pdf]

Table S1.Clinical trials of anti-fibrotic drugs associated with the Hh pathway discussed in this review

Drugs	NCT	N	Status	Phase	Disease	Study Start	Study completion
Vismodegib	NCT02648048	21	Completed	Phase 1	IPF	2016-01	2016-11
	NCT02593760	10	Completed	Phase 1	MF	2016-01	2017-07
Sonidegib	EudraCT No. 2011-005876-40	33	Completed	Phase 1	IHF	-	-
	NCT01787552	15	Completed	Phase 1b/2	MF	2013-05	2018-04
	NCT02151864	9	Completed	Phase 1	Cirrhosis	2014-07	2017-09
	NCT02086513	17	Terminated	Phase 1	cGVHD	2014-04	2016-12
Saridegib	NCT01371617	14	Completed	Phase 2	MF	2011-10	2012-08
Glasdegib	NCT03596567	18	Completed	Phase 1	Renal impairment	2018-05	2018-09
	NCT03627754	24	Completed	Phase 1	IHF	2018-11	2019-04
	NCT03415867	20	Unknown status	Phase 1b/2a	cGVHD	2018-01	2022-06
	NCT04111497	15	Terminated	Phase 1b/2a	cGVHD	2019-12	2023-08
	NCT02226172	21	Terminated	Phase 2	MF	2014-10	2018-01
Taladegib	NCT05817240	21	Completed	Phase 1	IPF	2023-05	2023-06
	NCT04968574	41	Completed	Phase 2	IPF	2021-08	2023-11
	NCT06422884	320	Ongoing	Phase 2	IPF, PPF	2024-09	2026-06
Itraconazole	NCT04783753	72	Completed	Phase 1	Chronic hepatitis B	2020-11	2021-07
	NCT00528190	35	Completed	Phase 4	CF	2007-10	2011-05
	NCT01576315	11	Completed	Phase 2	CF	2014-06	2015-11
	NCT06154447	159	Ongoing	Phase 1	CF	2023-12	2025-04
	NCT05049525	16	Terminated	Phase 2	IBD	2022-02	2024-02
Pirfenidone	NCT06267794	108	Completed	Phase 2	Cirrhosis	2015-06	2023-03
	NCT05542615	60	Recruiting	Phase 2	Cirrhosis	2019-08	2024-01
	NCT04461587	50	Completed	Phase 2	Pneumoconiosis	2020-08	2022-11
	NCT04928586	200	Not recruiting	Phase 4	CTD-ILD	2019-08	2025-06
	NCT05505409	120	Recruiting	Phase 4	CTD-ILD	2022-06	2025-12
	NCT03857854	152	Unknown status	Phase 3	Dm-ILD	2018-06	2021-05
	NCT00001959	21	Completed	Phase 2	FSGS	1999-12	2008-10
	NCT02496182	60	Unknown status	Phase 2/3	HP	2015-07	2017-01
	NCT04193592	50	Unknown	Phase 2	ILD	2019-12	2022-12

			status				
	NCT02821689	57	Unknown status	Phase 4	ILD	2016-07	2018-06
	NCT02808871	123	Completed	Phase 2	ILD	2017-04	2021-04
	NCT03208933	60	Completed	Phase 3	IPF	2017-10	2019-11
	NCT00080223	83	Completed	Phase 2	IPF	2003-08	2015-04
	NCT00662038	1058	Completed	Phase 3	IPF	2008-08	2016-02
	NCT02598193	89	Completed	Phase 4	IPF	2016-01	2017-05
	NCT02648048	21	Completed	Phase 1	IPF	2016-01	2016-11
	NCT02579603	105	Completed	Phase 4	IPF	2015-10	2017-01
	NCT06589921	24	Completed	Phase 1	IPF	2023-08	2023-09
	NCT06588517	12	Completed	Phase 1	IPF	2024-04	2024-04
	NCT04856111	48	Completed	Phase 4	IPF	2021-03	2022-11
	NCT02136992	160	Completed	Phase 2	IPF	2011-12	2013-12
	NCT00287716	435	Completed	Phase 3	IPF	2006-07	2008-11
	NCT00287729	344	Completed	Phase 3	IPF	2006-04	2008-11
	NCT02606877	37	Completed	Phase 4	IPF	2016-04	2017-03
	NCT01366209	555	Completed	Phase 3	IPF	2011-06	2014-02
	NCT02951429	177	Completed	Phase 2	IPF	2016-12	2020-08
	NCT03385668	7	Completed	Phase 2	PF	2018-01	2020-07
	NCT04607928	148	Unknown status	Phase 2	PF	2020-08	2022-06
	NCT01933334	63	Completed	Phase 2	SSc-ILD	2013-10	2014-09
	NCT03856853	144	Unknown status	Phase 3	SSc-ILD	2018-06	2021-05
Empagliflozin	NCT06149793	15	Recruiting	Phase 2/3	CF	2023-12	2025-12
	NCT05147090	106	Recruiting	Phase 4	Cirrhosis	2022-01	2025-12
	NCT06655480	50	Not recruiting	Phase 2	Myocardial fibrosis	2024-10	2026-12

Abbreviations: IPF: idiopathic pulmonary fibrosis; CF: cystic fibrosis; MF: myelofibrosis; cGVHD: chronic graft-versus-host disease; IHF: impaired hepatic function; PPF: progressive pulmonary fibrosis; NASH: non-alcoholic steatohepatitis; IBD: inflammatory bowel disease; PF: pulmonary fibrosis; ILD：interstitial lung disease; SSc-ILD: systemic sclerosis-related interstitial lung disease; CTD-ILD: connective tissue disease-related interstitial lung disease; Dm-ILD: dermatomyositis interstitial lung disease; FSGS: focal segmental glomerulosclerosis; HP: hypersensitivity pneumonitis.

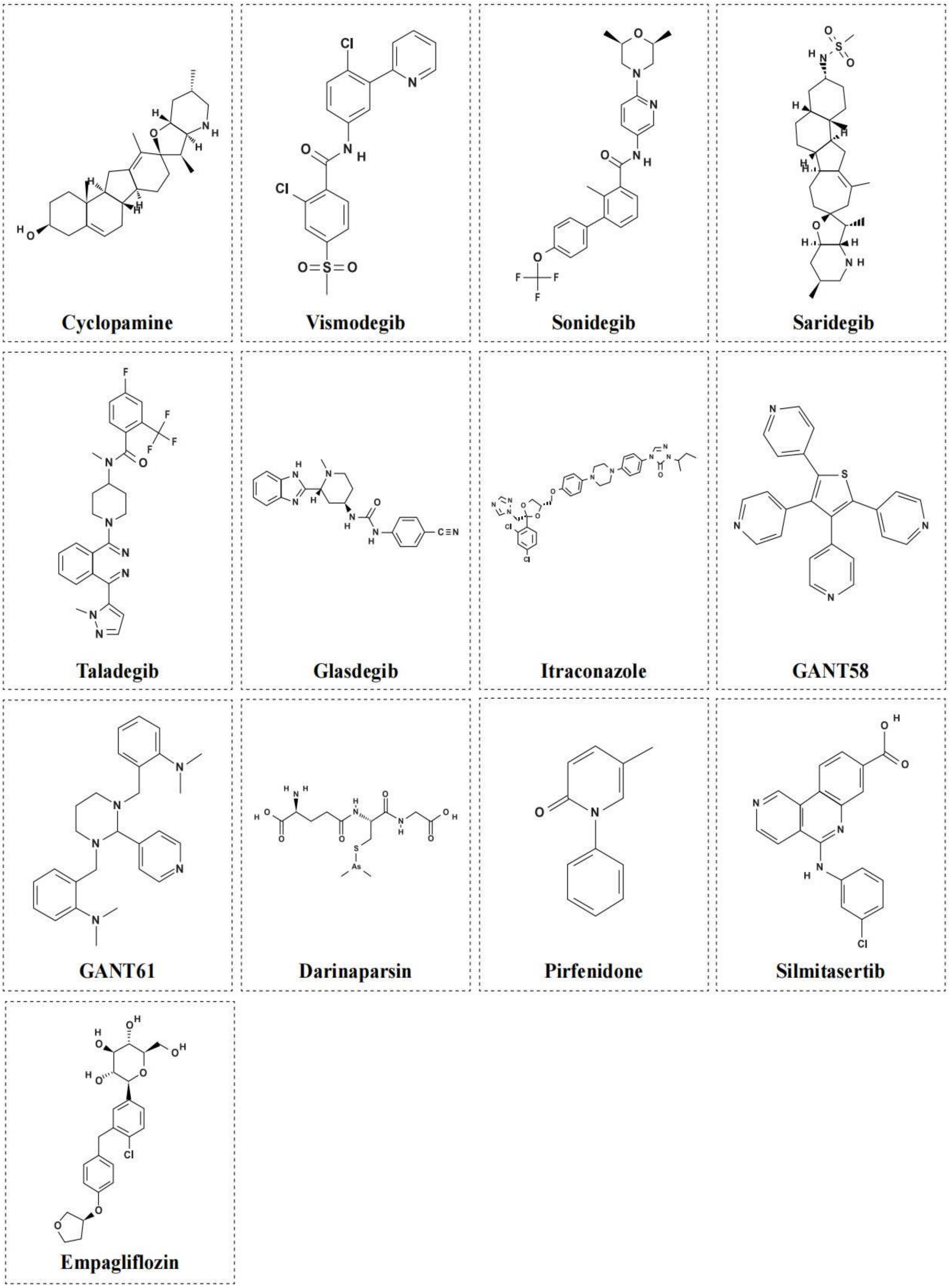

Figure S1. Chemical structures of anti-fibrotic drugs related to the Hh signaling pathway discussed in the review.
